# The relationship between children’s oral health behaviours and oral health-related quality of life: a cross-sectional study

**DOI:** 10.1186/s12903-023-03454-5

**Published:** 2023-10-13

**Authors:** Hanan Boodai, Mohamed ElSalhy, Aishah Alsumait, Jitendra Ariga, Marwan Al-Sharbati

**Affiliations:** 1 School Oral Health, Dental Administration, Ministry of Health, Kuwait City, Kuwait; 2 College of Dental Medicine, University of New England, Portland, ME USA; 3 Department of Social and Behaviour Al Sciences, Faculty of Public Health, Kuwait University, Kuwait City, Kuwait

**Keywords:** Oral health, Dental caries, Education, Behaviour, Students, OHRQoL

## Abstract

**Background:**

Understanding oral health behaviour s and their impact on Oral Health-Related Quality of Life (OHRQoL) may serve as an instrument to articulate the conventional oral health policy framework, thereby ameliorating the overall health of young individuals in the long term.

**Objective:**

The aim of this study was to investigate the relationship between children’s oral health behaviour s and Oral Health-Related Quality of Life in the capital governorate, Kuwait.

**Methods:**

A cross-sectional study involving 607 children aged 12–14 years, randomly selected from schools in Kuwait Capital Region. A validated Oral Health Behaviour s and OHRQoL Child Perception Questionnaires (CPQ_12-14_) was used to collect the data. Chi-square, t-tests, and ANOVA were used to examine the association between oral behaviour s and children’s OHRQoL.

**Results:**

About 52.2% of participants were males and the overall response rate was 93.8%. The mean ± SD for total OHRQoL impact was 3.1 ± 0.58, while the total mean for individual domains- for oral symptoms, functional limitations, emotional well-being and social well-being were 2.89 ± 0.63, 2.89 ± 0.72, 3.1 ± 0.91 and 3.4 ± 0.61, respectively. There was no significant difference in total OHRQoL impact score by frequency of last dental visit, flossing, use of mouth rinse or chewing gum (*p* > 0.05) but for the overall OHRQoL, frequency of soft drink intake was the only significant predictor associated with 0.2-unit decrease (B = -0.207, 95% CI, *p* = 0.002) in total OHRQoL scores.

**Conclusions:**

High frequency of soft drink consumption was related to poorer OHRQoL. Behaviour changing interventions based on OHRQoL inferences coupled with clinical intervention are needed.

**Supplementary Information:**

The online version contains supplementary material available at 10.1186/s12903-023-03454-5.

## Background

Oral health is defined as: “the state of the mouth, teeth and orofacial structures that enables individuals to perform essential functions such as eating, breathing and speaking, and encompasses psychosocial dimensions such as self-confidence, well-being and the ability to socialize and work without pain, discomfort and embarrassment” [1]. Oral health is a public health issue and justifiably seeks serious concern, especially among young children. Poor oral health is detrimental for children since it affects their nutrition, growth, and development in the long run. Further, childhood oral disease, if untreated, leads to pain, development of dentofacial anomalies and other serious health problems, such as severe toothache, dental abscess, destruction of bone, and spread of infection via the bloodstream [2].

Oral Behaviours are a set of routine practices for keeping the mouth clean and healthy, which substantially prevent oral diseases and maintain overall oral health. These behaviours include regular dental attendance, tooth brushing, regular use of fluoridated toothpaste, flossing, use of mouth rinse and gum chewing. As these behaviours can affect individual’s oral health status, they may play a role in impacting their Oral Health-Related Quality of Life (OHRQoL).

Oral Health-Related Quality of Life is defined as: “a subjective evaluation that depicts individual’s comfort while eating, sleeping and engaging in social interaction; his self-esteem; and his satisfaction with respect to their oral health” [2]. OHRQoL is purely based on personal information provided by individuals and exhibits the impact of oral health status on various aspects of life [3]. Measures of OHRQoL serve as indispensable tools in oral health studies as they can provide requisite data to be used by various flagship health programs and thus help in apportionment of various health resources [4]. It has been established that clinical indicators solely do not exhibit the full impact of oral conditions on the psychosocial well-being of a person [5]. For instance, in a study conducted among Libyan children from Benghazi, despite inadequate tooth brushing, a lower prevalence of dental caries was found as per WHO standards [6]. Therefore, besides the physical indicators, evaluating the social, psychological and economic implications can be used to identify population subgroups that need to be targeted for health promotion and disease prevention efforts [7]. Socio-dental approach amalgamating OHRQoL with the standard clinical measures comes in proximity to current concepts of health than the traditional standard approach [8, 9].

The Children Perception Questionnaire (CPQ_12-14_) is a commonly used self-administered Questionnaire that assess children’s OHRQoL [10]. It used Likert-type scales with score response options of “Never” = 4, “Once or twice” = 3, “Sometimes” = 2, “Often” = 1, and “Every day or almost every day” = 0 within a recall period of 3 months. The questionnaire consists of questions in four domains: oral symptoms, functional limitations, emotional well-being, and social well-being of children. Higher scores indicate better OHRQoL [10].

The aim of this study was to investigate the relationship between the student’s oral health practices and their OHRQoL. The study hypothesis is that poor oral health behaviour s among students are associated with low levels of OHRQoL.

## Methods

### Study design

This is a cross-sectional study conducted using self-administered structured questionnaires. The study protocol was approved by the Research Committee of the Faculty of Public Health, Kuwait University, Kuwait; and the Ethical Committee of the Ministry of Health, Kuwait (Protocol # 2019/1092). Parents/Guardians of participating students were informed by a letter which explained the nature of the study and a written consent form to participate in the study prior to data collection via questionnaires. Students who returned the consent form completed and signed were allowed to participate. A copy of the consent was shared with the parents. This process was partially supported by Kuwait’s School Oral Health Program.

### Study setting, data collection and sample size calculation

This study was conducted in the public schools of Capital Education/Health Region in Kuwait during the academic year 2019-2020. A multistage sampling method was used to get a random sample of equal proportions of both genders. The total number of students (both genders) in Grades 7 and 8 in the Capital Governorate as provided by the Ministry of Education was 3643 designated as the population of interest. The public intermediate schools in the capital area were shortlisted from list of all schools registered with the Ministry of Education (22 schools for girls and 20 schools for boys). Later, a random sampling of 16 schools (8 for boys and 8 for girls) was performed. All students from 7th and 8th Grade in those schools were invited to participate.

The estimated sample size was calculated by an online calculator of cross-sectional studies by Raosoft. Inc. [11]. The sample size was based on the total number of students at the beginning of the academic year 2019/2020, which was 3643 students within Capital governorate. The result of the calculation according to the above-mentioned formula was 348 students. However, as we anticipated to face withdrawal of some students from the study, or to get some incompletely filled questionnaire, and in order to reach sufficient statistical power, we took a larger sample size than the calculated one, to reach approximately 607 students divided equally between the two genders in this study. Approximately 80% power estimated using standard statistical operations such as confidence interval of 95%, margin of error 5% and response distribution 50%. The project was conducted by two dentists and a nurse in the School Oral Health Program team.

### Data collection instruments and procedures

Two self-administered questionnaires with demographic variables were used:

- *Oral Health (OH) Behaviours Questionnaire*: This included 8 questions based on routine behaviours, such as dental attendance, frequency of brushing, use of oral rinses, consumption of sugars, presence of toothache, etc. The format of the questions was adapted from the validated Health Behaviours in School- Aged Children (HBSC) survey [12, 13]. The questionnaire included two universal self-rating questions for evaluating individual’s perceived oral health with answers extending from “Excellent” = 4, “Very good” = 3, “Good” = 2, “Acceptable” = 1, to “Poor” = 0, and one question about the impact of oral health on overall well-being with responses “Not at all” = 4, “Very little” = 3, “Somewhat” = 2, “A lot” = 1 to “Very much” = 0.
- *Children Perception Questionnaire (CPQ*_*12-14*_*)*: A modified short form of the self-administered Children Perceptions Questionnaire was used to assess children’s OHRQoL [14]. It used Likert-type scales with score response options of “Never” = 4, “Once or twice” = 3, “Sometimes” = 2, “Often” = 1, and “Every day or almost every day” = 0 within a recall period of 3 months. This questionnaire consisted of questions in the form of domains namely- oral symptoms, functional limitations, emotional well-being, and social well-being of children, with 8 questions in each and few miscellaneous questions. The Arabic version of the CPQ_12-14_ questionnaire was previously translated and validated in Kuwait [15].

### Statistical analysis

Data was analyzed using SPSS 22.0 software (IBM Corp., Armonk, N.Y., USA). The Shapiro-Wilks test was used for the determination of continuous variables for normal distribution while frequency and percentage distribution depicted the categorical variables such as oral health behaviour s in terms of score values (e.g. 0, 1, 2, 3, etc.) for every response. The CPQ_12-14_ OHRQoL scores for each domain and overall OHRQoL were determined by aggregating all derived outcomes to factors in the corresponding domains or by summation of such outcomes represented as the whole questionnaire. The overall lower score indicates superior OHRQoL and vice-versa. While the Chi-square examined gender difference in oral health between various behaviours, t-test investigated any gender difference in each domain as well as overall OHRQoL scores. ANOVA test assessed difference in domains and overall OHRQoL scores among various behaviours. Post hock and Bonferroni test were applied to find significant difference within the groups.

Further, the linear regression analyses identified the predictors of OHRQoL domains and total scores. Dependent variables included in the regression analysis were children’s comfort while eating, sleeping and engaging in social interaction, while the independent variables were gender (ref. category: male), last dental visit (ref. category: less than 1 year), brushing frequency (ref. category: more than once a day), use of mouth rinse (ref. category: more than once a day), flossing frequency (ref. category: more than once a day), gum chewing (ref. category: more than once a day), frequency of sugar intake (ref. category: more than once a day) and frequency of soft drinks consumption (ref. category: more than once a day). The level of significance for all quantitative tests was be set at *p*≤ 0.05.

## Results

A total of 607 students (with 52.2% males), participated in the study with a response rate of 93.8%. About 60% of students had visited the dentist within the previous year and 23.4% between 1-2 years (Table 1). About 48.7% of participant brush their teeth more than once a day and only 15.4% rarely or never. More than half of the participants (54.1%) consume sugary foods/drinks more than once a day and almost 66.5% of students drink soft drinks at least once a day. Nearly 43% of children used chewing gum more than once a day. Out of all examined behaviour s, five of them showed significant association with gender (*p*<0.05). These behaviour s were dental visits, toothbrushing, gum chewing, sugar intake and frequency of soft drinks consumption (Table 1).

**Table 1:** Oral health practices and symptoms among 12–14-year-old students by gender (*N* = 607), Kuwait, 2020

Behaviours (N)	Girls		Boys		Total		*p*-value
	**N**	**%**	**N**	**%**	**N**	**%**	
**1. Last dental visit**							
< 1 year	192	66.2	173	54.6	365	60.1	**0.011**
1–2 years	60	20.7	82	25.9	142	23.4	
> 2 two years	38	13.1	62	19.6	100	16.5	
Total	290	100	317	100	607	100	
**2. Experienced dental pain**							
Never	41	14.1	57	18.0	98	16.2	0.379
Less than a year	180	62.1	181	57.3	361	59.6	
1–2 years	42	14.5	41	13.0	83	13.7	
More than 2 years	27	9.3	37	11.7	64	10.6	
Total	290	100	316	100	606	100	
**3. Tooth brushing frequency**							
More than once a day	186	64.4	107	34.2	293	48.7	** < 0.001**
Once a day	83	28.7	133	42.5	216	35.9	
Rarely/Never	20	6.9	73	23.3	93	15.4	
Total	289	100	313	100	602	100	
**4. Dental flossing**							
More than once a day	17	5.9	21	6.7	38	6.3	0.172
Once a day	63	22.0	50	16.0	113	18.8	
Rarely/Never	207	72.1	242	77.3	449	74.8	
Total	287	100	313	100	600	100	
**5. Use of mouth rinse**							
More than once a day	61	21.4	76	24.9	137	23.2	0.512
Once a day	79	27.7	75	24.6	154	26.1	
Rarely/Never	145	50.9	154	50.5	299	50.7	
Total	285	100	305	100	590	100	
**6. Use of chewing gum**							
More than once a day	160	55.6	96	31.1	256	42.9	** < 0.001**
Once a day	75	26.0	113	36.6	188	31.5	
Rarely/Never	53	18.4	100	32.4	153	25.6	
Total	288	100	309	100	597	100	
**7. Frequency of sugar intake**							
More than once a day	163	57.2	161	51.3	324	54.1	**0.002**
Once a day	106	37.2	108	34.4	214	35.7	
Rarely/Never	16	5.6	45	14.3	61	10.2	
Total	285	100	314	100	599	100	
**8. Frequency of Soft Drinks**							
More than once a day	73	25.4	122	39.0	195	32.5	**0.002**
Once a day	108	37.6	96	30.7	204	34.0	
Rarely/Never	106	36.9	95	30.4	201	33.5	
Total	287	100	313	100	600	100	

Table 2 shows the self-rating of oral health where more than two-thirds (70%) of children perceived their oral health to be excellent, very good and good while only 30% described it to be fair or poor. About three quarters (72.8%) of the students indicated that their oral health condition does not affect their overall life at all or affects very little, while 27.2% reported otherwise (Table 2).

**Table 2:** Self-rating of oral health and its impact responses among 12–14-year-old students (*N* = 607), Kuwait, 2020

**1. Would you say the health of your teeth, lips, jaws, and mouth is:**						
	**Excellent**	**Very good**	**Good**	**Fair**	**Poor**	**Total**
**Percentage**	14.8	28.7	26.5	19.8	10.2	100
**2. How much does the condition of your teeth, lips, jaws or mouth affect your life overall?**						
	**Not at all**	**Very little**	**Sometimes**	**A lot**	**Very much**	**Total**
**Percentage**	38.8	34.8	18.9	5.5	2.8	100

The mean (SD) for total OHRQoL impact was 3.1 (0.58), while the total mean for the domains were: 2.89 (0.63) for oral symptoms, 2.89 (0.72) for functional limitations, 3.1 (0.91) for emotional well-being, and 3.4 (0.61) for social well-being respectively. Girls had significantly higher mean oral symptoms scores compared to boys (2.8 vs. 2.9; *p*<0.001) with no difference in all other OHRQoL domains or total OHRQoL (Table 3).

**Table 3:** The mean and standard deviation (SD) values for each domain in both genders of 12–14 years aged students (*N* = 607), Kuwait, 2020

Domain	Girls		Boys		Both Genders		*p*-value*
	**Mean**	**SD**	**Mean**	**SD**	**Mean**	**SD**	
**Oral symptoms**	2.8	0.7	3.0	0.6	2.9	0.6	** < 0.001**
**Functional limitations**	2.9	0.7	2.9	0.7	2.9	0.7	0.205
**Emotional well-being**	3.1	0.9	3.1	0.9	3.1	0.9	0.845
**Social well-being**	3.4	0.6	3.4	0.6	3.4	0.6	0.694
**Total OHRQoL score**	3.1	0.6	3.2	0.5	3.1	0.6	0.145

^*^Variable with significant *p*-values are in bold

Another set of supplementary tables (Additional file 1: Appendix I) categorically enumerates the different oral behaviours/symptoms (Table S1), functional limitations (Table S2), emotional well-being (Table S3), social well-being (Table S4) and total OHRQoL (Table S5) as parameters to assess the overall OHRQoL among the young population of Kuwait. Students who consume soft drinks once a day or more reported significantly lower scores in all OHRQoL domains as well as the total OHRQoL impact scores than those who rarely/never have soft drinks a day (*p*<0.05). Furthermore, there is a significant difference between mean scores of students who were having soft drinks more than once a day and those who had it once a day (*p*<0.05).

There was a significant difference in the mean oral symptoms (*p*<0.001), functional limitations (*p*=0.024), emotional well-being (*p*=0.005) and OHRQoL impact scores (*p*<0.001) according to sugar consumption frequency. Students who consumed sugars more than once a day had significantly higher oral symptoms, functional limitations, emotional well-being and OHRQoL impact scores as compared to students having lower frequency of sugar consumption. No significant difference was observed between students who rarely consumed sugar and those who had sugar once a day (*p*>0.05).

Students who consume soft drinks once a day or more reported significantly lower scores in all OHRQoL domains as well as the total OHRQoL impact scores than those who rarely/never have soft drinks a day (*p*<0.05). Furthermore, there is a significant difference between mean scores of students who were having soft drinks more than once a day and those who had it once a day (*p*<0.05).

There was no significant difference in individual domain or total OHRQoL impact score by frequency of last dental visit, flossing, use of mouth rinse or use of chewing gum. Tables (S1, S2, S3, S4 and S5) illustrate the relationship between OHRQoL domains and oral health behaviour s.

After adjusting for gender and oral health behaviour s in the linear regression model, the frequency of brushing and the frequency of soft drink intake were the only significant predictors of oral symptoms (Table 4). Brushing once a day or rarely, were associated with a 0.1-unit (B=-0.134, 95% CI: - 0.250- -0.018, *p*=0.024) and nearly 0.3-unit (B=-0.27, 95% CI: -0.438- -0.105, *p*=0.001) lower oral symptom scores (worse symptoms) compared to students who brushed more often. The soft drinks intake above once a day limit was associated with a 0.2-unit lower oral symptom score as compared to students who rarely uses soft drinks a day (B=-0.192, 95% CI: -0.326- -0.058, *p*=0.005). The frequency of soft drinks consumption was the only significant predictors of functional limitations after adjusting for gender and oral health behaviour s (Table 4). Drinking soft drinks more once a day was associated with 0.04-unit decrease in functional limitations scores compared to children who rarely uses soft drinks a day (B=-0.287, 95% CI: -0.443- -0.13, *p*<0.001). For emotional symptoms, none of the behaviours were significantly correlated with Emotional well-being. The frequency of soft drinks intake was the only significant predictors of social symptoms after adjusting for gender and oral health behaviours (Table 4).

**Table 4:** Oral health behaviours as predictors of OHRQoL in 12–14 years aged students (*N* = 607), Kuwait, 2020^a^

Variables	Coefficient	SE	*p*-value*	95% CI	
				**Lower limit**	**Upper limit**
**Outcome variable: Oral Symptoms**					
Constant	2.091	0.402	0.000	1.302	2.88
Brushing once a day	-0.134	0.059	**0.024**	-0.250	-0.018
Brushing rarely or never	-0.271	0.085	**0.001**	-0.438	-0.105
Frequency of Soft Drinks consumption more than once a day	-0.192	0.068	**0.005**	-0.326	-0.058
*R*^*2*^ = 0.165; Adjusted *R*^*2*^ = 0.089; Durbin-Watson = 2.014					
**Outcome variable: Functional Limitations**					
Constant	2.606	0.468	0.000	1.688	3.525
Frequency of Soft Drinks consumption more than once a day	-0.287	0.080	0.000	-0.443	-0.130
*R*^*2*^ = 0.059; Adjusted *R*^*2*^ = 0.042; Durbin-Watson = 2.014					
**Outcome variable: Emotional well-being**					
Constant	1.636	0.601	**0.007**	0.455	2.817
*R*^*2*^ = 0.042; Adjusted *R*^*2*^ = 0.025; Durbin-Watson = 2.065					
**Outcome variable: Social well-being**					
Constant	2.505	0.399	0.000	1.720	3.289
Frequency of Soft Drinks consumption more than once a day	-0.176	0.067	**0.009**	-0.308	-0.043
*R*^*2*^ = 0.047; Adjusted *R*^*2*^ = 0.029; Durbin-Watson = 1.961					
**Outcome variable: Total OHRQoL Score**					
Constant	2.211	0.393	0.000	1.438	2.984
Frequency of Soft Drinks consumption more than once a day	-0.207	0.067	**0.002**	-0.337	-0.076
*R*^*2*^ = 0.074; Adjusted *R*^*2*^ = 0.055; Durbin-Watson = 2.066					

^a^Predictors examined in the models: gender (reference: male), Brushing frequency (reference category: more than once a day), frequency of sugar intake (reference category: more than once a day) and frequency of Soft Drink intake (reference category: more than once a day)

^*^Variables with significant *p*-values are in bold

The consumption of soft drinks more than once a day was associated with nearly 0.2 unit lower in social symptoms scores compared to students who rarely use soft drinks a day (B=-0.176, 95% CI: -0.308- - 0.0.043, *p*=0.009). For the overall OHRQoL, frequency of soft drink intake was the only significant predictor (Table 4). The intake of soft drinks more than once a day was associated with 0.2-unit lower in total OHRQoL scores compared to children who rarely uses soft drinks a day (B=-0.207, 95% CI: -0.337- -0.076, *p*=0.002).

## Discussion

Identifying specific oral health behaviours that affect and predict OHRQoL is important to guide various oral health education and promotion programs. This cross-sectional study examined various oral behaviours and their relationship with OHRQoL among students. Out of all examined behaviours, frequency of soft drinks consumption more than once a day was a significant predictor of OHRQoL among 12-14 years old students. The frequency of soft drink consumption more than once a day was correlated with oral symptoms, functional limitations, social well-being, and total OHRQoL scores. In addition, about 40% of children included in this study consumed soft drinks more than once a day, a behaviour reported to cause early caries [16–18].

In this study, data were gathered through face-to-face interviews to eschew any data loss, minimize information bias, and enhance the accuracy of data [19]. The response rate was 93.8% which might be due to the anonymous use of the instruments (i.e. CPQ_12-14_ questionnaire) ensuring participant confidentiality. Although due to the limitations in the present study design, being a cross-sectional one, inferences so obtained cannot impress a direct impact on the OHRQoL, yet the manifestation of high soft drinks consumption by children does offer a reconsideration towards their present understanding of oral health. The findings of the present study were also in agreement with several studies conducted among Kuwaiti school children [15–18].

It has also been ruled in that since most of the sweet beverages seem to contain acid and regular sugar, the development of both dental erosion and caries are likely to have been positively influenced by high soft drinks drinking [18]. Although this correlation substantiates the findings that high consumption of soft drinks among youngsters was related to poor oral health as compared to those with lower consumption of the same, yet, it is not conceivable to account soft drinks consumption as the sole cause of the deteriorating oral health [20, 21]. However, it was also observed that lifestyle changes, with regimented soft drinks consumption, have the potential to positively affect the oral health of young individuals [22]. Consequently, the present study supports the contemplation that keeping an account of soft drinks consumption by students using educative learning could serve as an instrument for predicting such behaviour.

The findings of current study bring our attention towards the importance of adopting the practices which can improve consciousness among caretakers and students for maintaining proper oral hygiene from early stage convalescing overall oral health. A more comprehensive model, on the contrary, such as the common risk factor approach may bring about better outcomes in changing the soft drinks consumption behaviour in children than a conventional oral health education approach [23]. This approach targets the anomalous consumption behaviour by taking into consideration its impacts on chronic diseases such as diabetes, obesity, dental caries, etc. Educative methods play the same role quite differently. For example, children can be provided with friendly environment to ensure healthful physical activity and be encouraged to participate in outdoor sports. For this purpose, in addition to the governmental efforts in providing appropriate physical environments through creation of walkways in different municipalities, suitable school playgrounds, public sport yards, private sector under corporate social responsibility initiative can also contribute to sponsoring gymnasiums, sports stadiums, sports gears, etc. Government initiatives to promote oral health could also be stepped up to encourage parents to take responsibility for their child's health by monitoring their child's oral health at home, as well as bringing their child to see the dentist right from a young age. Additionally, the Government institutions can enact laws and regulations or amend the policies for taxation of sugary drinks and regulating the sale of sweetened products in and around school premises [24]. In schools, dental experts can assist the teachers to come up with the proper revision to current health curriculums that could promote a healthy lifestyle, through nutritious and balanced diets, followed by fun games to entice children towards a better quality of life. Similarly, parents can also be educated about various routine preventive measures, such as regular dental visits, proper toothbrushing, mouth rinsing, besides balanced intake of sugary products by their children. Inculcating a subjective perception towards oral health especially by the youngsters, is very crucial as it directly influences their long-term oral health behaviours. Our findings in the study suggest that dental clinicians should also contemplate subjective measures like quality of life along with their clinic assessment in treatment planning [25]. The OHRQoL measures have the potential to stipulate insights into how oral conditioning emphasize various physical and psychological aspects of everyday life among children. Consequently, they can complement traditional or professionally determined outcome measures for the assessment of orthodontic treatment needs in addition to prioritization of care to those who need it most.

To our knowledge, this study is one of the first studies to explore the relationship between oral health behaviour s and OHRQoL among 12-14 years students in Kuwait. Another strength of the study is the randomness of the sample, the high response rate (93.8%), as well as large sample size (607 students). Furthermore, the present study used a strong, validated, and reliable questionnaire that had been previously used with similar populations [18]. All these points are positively reflected in the capability of the study generalization. On the other hand, the main weak point in this study was attributed to the nature of the study design, being a cross-sectional one, so the cause-effect relations cannot be established, however, it could help in generating valuable hypotheses, and provides insights into potentially valuable associations of different parameters. Another limitation was the use of a self- administered questionnaire which is subjected to incomplete responses and reporting, and recall bias. In addition, this study did not include caries incidence of the study sample in consideration in the relations investigated.

## Conclusion

Out of all examined behaviours, frequency of soft drinks consumption more than once a day was a significant predictor of OHRQoL among 12-14 years old students. Comprehensive health promotion approaches like the common risk factor approach targeting sugar consumption as policy level change can opt to discourage the consumption of sugar; the teachers and dental experts should engage caretakers with regular counselling, parents should be adequately vigilant to fulfil their responsibility for maintaining the overall well-being of their children and religious adoption of pragmatic preventive measures must be encouraged to improve their OHRQoL perpetually.

### Supplementary Information

**Additional file 1: Table S1.** Oral health behaviors/symptoms as a measure of OHRQoL in both genders of 12-14 years aged students (*N*=607), Kuwait, 2020. **Table S2.** Functional Limitations as a measure of OHRQoL in both genders of 12-14 years aged students5653405244411500 (*N*=607), Kuwait, 2020. **Table S3.** Emotional well-being as a measure of OHRQoL in both genders of 12-14 years aged students (*N*=607), Kuwait, 2020. **Table S4.** Social well-being as a measure of OHRQoL in both genders of 12-14 years aged students (*N*=607), Kuwait, 2020. **Table S5.** Total OHRQoL scores of all the oral behaviours in both genders of 12-14 years aged students (*N*=607), Kuwait, 2020.

## Data Availability

The datasets during and/or analyzed during the current study available from the corresponding author on reasonable requests.

## Abbreviations

OH: Oral health
OHRQoL: Oral health related quality of life
WHO: World health organization
USDHHS: United states department of health
DMFT: Decayed, missing due to caries, and filled teeth
DMFS: Decayed, missing due to caries, and filled surfaces
COI: Cost of Illness
HBSC: Health behaviours in school-aged children
CPQ12-14: Children perception questionnaire
ANOVA: Analysis of variance
SD: Standard Deviation
CI: Confidence interval
SE: Standard Error
