# Supplementary material for: The relationship between children’s oral health behaviours and oral health-related quality of life: a cross-sectional study
Source: BMC Oral Health. 2023 Oct 13;23:757. doi: 10.1186/s12903-023-03454-5 (PMC10576284; doi:10.1186/s12903-023-03454-5)
Supplement: Supplementary file 1 — Additional file 1: Table S1. Oral health behaviors/symptoms as a measure of OHRQoL in both genders of 12-14 years aged students (N=607), Kuwait, 2020. Table S2. Functional Limitations as a measure of OHRQoL in both genders of 12-14 years aged students5653405244411500 (N=607), Kuwait, 2020. Table S3. Emotional well-being as a measure of OHRQoL in both genders of 12-14 years aged students (N=607), Kuwait, 2020. Table S4. Social well-being as a measure of OHRQoL in both genders of 12-14 years aged students (N=607), Kuwait, 2020. Table S5. Total OHRQoL scores of all the oral behaviours in both genders of 12-14 years aged students (N=607), Kuwait, 2020. [file 12903_2023_3454_MOESM1_ESM.docx]

**Table S1. Oral health behaviors/symptoms as a measure of OHRQoL in both genders of 12-14 years aged students (N=607), Kuwait, 2020**

| **OHRQoL Score** | | | | | | | |
| --- | --- | --- | --- | --- | --- | --- | --- |
| **Oral Behaviors/symptoms** | **Girls** | | **Boys** | | **Both Genders** | | **p-value** |
|  | **Mean** | **SD** | **Mean** | **SD** | **Mean** | **SD** |  |
| **1. Last dental visit** |  |  |  |  |  |  | 0.120 |
| < 1 year | 3.0403 | .54534 | 2.8660 | .66142 | 2.9587 | .60780 |  |
| 1-2 years | 2.8659 | .68738 | 2.6766 | .70777 | 2.7574 | .70288 |  |
| > 2 two years | 2.9259 | .65761 | 2.8074 | .55982 | 2.8519 | .59774 |  |
| **2. Pain** |  |  |  |  |  |  | **0.012** |
| Never | 3.2339 | .60185 | 3.2844 | .50702 | 3.2626^a^ | .54724 |  |
| Less than a year | 2.9088 | .55539 | 2.6202 | .68070 | 2.7649^b^ | .63675 |  |
| 1-2 years | 3.0894 | .56065 | 2.8803 | .43438 | 2.9875^c^ | .51086 |  |
| More than 2 years | 3.0046 | .79550 | 2.9228 | .56395 | 2.9556 ^c^ | .66106 |  |
| **3. Brushing Frequency** |  |  |  |  |  |  | **<0.001** |
| More than once a day | 3.0741 | .56265 | 2.9507 | .62174 | 3.0309^a^ | .58587 |  |
| Once a day | 2.8861 | .59638 | 2.7874 | .66165 | 2.8256^b^ | .63757 |  |
| Rarely/Never | 2.4815 | .67673 | 2.6190 | .67270 | 2.5948^c^ | .67143 |  |
| **4. Dental flossing** |  |  |  |  |  |  | 0.560 |
| More than once a day | 2.9150 | .57404 | 2.6167 | .81289 | 2.7538 | .71967 |  |
| Once a day | 3.0565 | .64903 | 2.8068 | .59924 | 2.9491 | .63742 |  |
| Rarely/Never | 2.9739 | .58271 | 2.8193 | .65401 | 2.8905 | .62627 |  |
| **5. Use of mouth rinse** |  |  |  |  |  |  | 0.439 |
| More than once a day | 2.9529 | .73071 | 2.7825 | .63714 | 2.7538 | .71967 |  |
| Once a day | 2.9444 | .58945 | 2.8164 | .57028 | 2.9491 | .63742 |  |
| Rarely/Never | 3.0403 | .52132 | 2.7980 | .71623 | 2.8905 | .62627 |  |
| **6. Use of chewing gum** |  |  |  |  |  |  | 0.851 |
| More than once a day | 2.9708 | .58035 | 2.7814 | .61885 | 2.8994 | .60097 |  |
| Once a day | 3.0172 | .64479 | 2.8308 | .65727 | 2.9043 | .65695 |  |
| Rarely/Never | 3.0022 | .58478 | 2.8176 | .68346 | 2.8834 | .65394 |  |
| **7. Frequency of sugar intake** |  |  |  |  |  |  | **0.011** |
| More than once a day | 2.9047 | .60215 | 2.7287 | .64263 | 2.8170^a^ | .62786 |  |
| Once a day | 3.1155 | .55145 | 2.9233 | .62828 | 3.0204^b^ | .59708 |  |
| Rarely/Never | 3.1508 | .54886 | 2.8140 | .74957 | 2.8967 | .71605 |  |
| **8. Frequency of Soft Drinks** |  |  |  |  |  |  | **0.004** |
| More than once a day | 2.8032 | .70658 | 2.6398 | .70860 | 2.7013^a^ | .71037 |  |
| Once a day | 2.9989 | .50499 | 2.8901 | .53083 | 2.9468^b^ | .51904 |  |
| Rarely/Never | 3.1111 | .56954 | 2.9331 | .67145 | 3.0291^c^ | .62329 |  |

**^ Within columns, variables with superscripts (^a,b,c^) are statistically significant at 0.05 level (t-test)**

*** Variables with significant p-values are in bold**

**Table S2. Functional Limitations as a measure of OHRQoL in both genders of 12-14 years aged students (N=607), Kuwait, 2020**

| **Functional Limitation of OHRQoL** | | | | | | | |
| --- | --- | --- | --- | --- | --- | --- | --- |
| **Oral Behaviors/symptoms** | **Girls** | | **Boys** | | **Both Genders** | | **p-value** |
|  | **Mean** | **SD** | **Mean** | **SD** | **Mean** | **SD** |  |
| **1. Last dental visit** |  |  |  |  |  |  | 0.540 |
| < 1 year | 2.9044 | .71492 | 2.8792 | .75110 | 2.8924 | .73145 |  |
| 1-2 years | 3.0263 | .69207 | 2.7650 | .76656 | 2.8753 | .74472 |  |
| > 2 two years | 2.9333 | .80966 | 2.9139 | .59383 | 2.9211 | .67715 |  |
| **2. Pain** |  |  |  |  |  |  | **0.004** |
| Never | 3.1966 | .66102 | 3.2862 | .57212 | 3.2482^a^ | .60950 |  |
| Less than a year | 2.8168 | .74308 | 2.7311 | .75338 | 2.7733^b^ | .74847 |  |
| 1-2 years | 3.1375 | .59663 | 2.7807 | .66679 | 2.9637 ^c^ | .65280 |  |
| More than 2 years | 2.9933 | .69635 | 2.9099 | .64262 | 2.9435 ^c^ | .66045 |  |
| **3. Brushing Frequency** |  |  |  |  |  |  | 0.281 |
| More than once a day | 2.9857 | .66183 | 2.9256 | .69024 | 2.9634 | .67188 |  |
| Once a day | 2.8457 | .79790 | 2.8867 | .76131 | 2.8708 | .77405 |  |
| Rarely/Never | 2.8246 | .89144 | 2.7066 | .71573 | 2.7315 | .75231 |  |
| **4. Dental flossing** |  |  |  |  |  |  | 0.446 |
| More than once a day | 2.8437 | .51449 | 2.7583 | .87772 | 2.7963 | .73042 |  |
| Once a day | 2.8468 | .87845 | 2.6522 | .82912 | 2.7639 | .85929 |  |
| Rarely/Never | 2.9691 | .67752 | 2.9008 | .68745 | 2.9316 | .68305 |  |
| **5. Use of mouth rinse** |  |  |  |  |  |  | 0.202 |
| More than once a day | 2.8060 | .77769 | 2.7877 | .79118 | 2.7963 | .73042 |  |
| Once a day | 2.9560 | .72026 | 2.8266 | .70788 | 2.7639 | .85929 |  |
| Rarely/Never | 3.0024 | .68061 | 2.8915 | .72362 | 2.9316 | .68305 |  |
| **6. Use of chewing gum** |  |  |  |  |  |  | 0.304 |
| More than once a day | 2.9150 | .71850 | 2.7456 | .71906 | 2.8496 | .72199 |  |
| Once a day | 3.0394 | .64246 | 2.8303 | .74738 | 2.9126 | .71351 |  |
| Rarely/Never | 2.8467 | .81639 | 3.0053 | .69020 | 2.9502 | .73752 |  |
| **7. Frequency of sugar intake** |  |  |  |  |  |  | **0.024** |
| More than once a day | 2.8300 | .74226 | 2.7652 | .71286 | 2.7969^a^ | .72692 |  |
| Once a day | 3.0550 | .67008 | 2.9559 | .71057 | 3.0057^b^ | .69062 |  |
| Rarely/Never | 3.1458 | .71718 | 2.9612 | .79502 | 3.0113 | .77303 |  |
| **8. Frequency of Soft Drinks** |  |  |  |  |  |  | **0.017** |
| More than once a day | 2.7315 | .78998 | 2.6751 | .76728 | 2.6965^a^ | .77436 |  |
| Once a day | 2.9783 | .69051 | 2.8670 | .66153 | 2.9244^b^ | .67717 |  |
| Rarely/Never | 3.0367 | .67319 | 3.0842 | .68645 | 3.0593^c^ | .67817 |  |

**^ Within columns, variables with superscripts (^a,b,c^) are statistically significant at 0.05 level (t-test)**

*** Variables with significant p-values are in bold**

**Table S3. Emotional well-being as a measure of OHRQoL in both genders of 12-14 years aged students (N=607), Kuwait, 2020**

| **Emotional well-being of OHRQoL** | | | | | | | |
| --- | --- | --- | --- | --- | --- | --- | --- |
| **Oral Behaviors/symptoms** | **Girls** | | **Boys** | | **Both Genders** | | **p-value** |
|  | **Mean** | **SD** | **Mean** | **SD** | **Mean** | **SD** |  |
| **1. Last dental visit** |  |  |  |  |  |  | 0.220 |
| < 1 year | 3.1263 | .87642 | 3.1406 | .92622 | 3.1330 | .89886 |  |
| 1-2 years | 3.2298 | .85517 | 3.0299 | .89117 | 3.1160 | .87829 |  |
| > 2 two years | 2.8921 | 1.16175 | 3.2579 | .91185 | 3.1172 | 1.02471 |  |
| **2. Pain** |  |  |  |  |  |  | 0.175 |
| Never | 3.3778 | .93295 | 3.4193 | .87316 | 3.4014 | .89461 |  |
| Less than a year | 3.0815 | .90418 | 3.0033 | .97834 | 3.0424 | .94140 |  |
| 1-2 years | 3.1534 | .81241 | 3.2102 | .60166 | 3.1800 | .71751 |  |
| More than 2 years | 2.9060 | 1.06095 | 3.2469 | .85123 | 3.1039 | .95175 |  |
| **3. Brushing Frequency** |  |  |  |  |  |  | 0.321 |
| More than once a day | 3.1409 | .90701 | 3.2233 | .90765 | 3.1706 | .90648 |  |
| Once a day | 3.1364 | .95581 | 3.0941 | .93780 | 3.1106 | .94271 |  |
| Rarely/Never | 2.8025 | .80174 | 3.0474 | .89061 | 2.9961 | .87397 |  |
| **4. Dental flossing** |  |  |  |  |  |  | 0.983 |
| More than once a day | 3.0980 | .82578 | 3.1222 | .83296 | 3.1111 | .81817 |  |
| Once a day | 3.1315 | .90186 | 3.1014 | .90626 | 3.1184 | .89957 |  |
| Rarely/Never | 3.1083 | .93290 | 3.1452 | .92088 | 3.1281 | .92556 |  |
| **5. Use of mouth rinse** |  |  |  |  |  |  | 0.357 |
| More than once a day | 3.1037 | .91804 | 3.1667 | .88575 | 3.1111 | .81817 |  |
| Once a day | 3.2496 | .75966 | 3.1877 | .76250 | 3.1184 | .89957 |  |
| Rarely/Never | 3.0603 | .98990 | 3.0741 | 1.00481 | 3.1281 | .92556 |  |
| **6. Use of chewing gum** |  |  |  |  |  |  | 0.963 |
| More than once a day | 3.1024 | .89938 | 3.0035 | .92728 | 3.1429 | .81578 |  |
| Once a day | 3.1320 | .91273 | 3.1331 | 1.00281 | 3.0645 | .90958 |  |
| Rarely/Never | 3.1342 | .98670 | 3.2790 | .77254 | 3.2253 | .85762 |  |
| **7. Frequency of sugar intake** |  |  |  |  |  |  | **0.005** |
| More than once a day | 2.9580 | .96422 | 3.1096 | .91731 | 3.0328^a^ | .94290 |  |
| Once a day | 3.3100 | .83973 | 3.2031 | .86272 | 3.2568^b^ | .85078 |  |
| Rarely/Never | 3.3819 | .68128 | 3.1010 | .99837 | 3.1759 | .92743 |  |
| **8. Frequency of Soft Drinks** |  |  |  |  |  |  | **0.01** |
| More than once a day | 2.8986 | 1.06876 | 2.9933 | .97926 | 2.9580^a^ | 1.01172 |  |
| Once a day | 3.2179 | .76490 | 3.1929 | .85648 | 3.2062^b^ | .80712 |  |
| Rarely/Never | 3.1547 | .93423 | 3.2695 | .85053 | 3.2075^c^ | .89616 |  |

**^ Within columns, variables with superscripts (^a,b,c^) are statistically significant at 0.05 level (t-test)**

*** Variables with significant p-values are in bold**

**Table S4. Social well-being as a measure of OHRQoL in both genders of 12-14 years aged students (N=607), Kuwait, 2020**

| **Social well-being of OHRQoL** | | | | | | | |
| --- | --- | --- | --- | --- | --- | --- | --- |
| **Oral Behaviors/Symptoms** | **Girls** | | **Boys** | | **Both Genders** | | **p-value** |
|  | **Mean** | **SD** | **Mean** | **SD** | **Mean** | **SD** |  |
| **1. Last dental visit** |  |  |  |  |  |  | 0.956 |
| < 1 year | 3.4502 | .58257 | 3.4127 | .61353 | 3.4321 | .59709 |  |
| 1-2 years | 3.4266 | .68195 | 3.3930 | .73190 | 3.4079 | .70772 |  |
| > 2 two years | 3.4595 | .54207 | 3.5056 | .52405 | 3.4878 | .52871 |  |
| **2. Pain** |  |  |  |  |  |  | 0.234 |
| Never | 3.6083 | .62195 | 3.6186 | .58423 | 3.6141 | .59758 |  |
| Less than a year | 3.3967 | .62436 | 3.3426 | .66971 | 3.3694 | .64724 |  |
| 1-2 years | 3.4603 | .51114 | 3.3739 | .57668 | 3.4187 | .54198 |  |
| More than 2 years | 3.4936 | .48815 | 3.5901 | .45786 | 3.5503 | .46915 |  |
| **3. Brushing Frequency** |  |  |  |  |  |  | 0.598 |
| More than once a day | 3.4423 | .61739 | 3.5359 | .53488 | 3.4759 | .58985 |  |
| Once a day | 3.4811 | .49728 | 3.3635 | .68555 | 3.4072 | .62362 |  |
| Rarely/Never | 3.3186 | .79998 | 3.3876 | .63876 | 3.3735 | .67013 |  |
| **4. Dental flossing** |  |  |  |  |  |  | 0.740 |
| More than once a day | 3.4657 | .50609 | 3.4875 | .50889 | 3.4775 | .50064 |  |
| Once a day | 3.3903 | .65823 | 3.4184 | .55888 | 3.4026 | .61392 |  |
| Rarely/Never | 3.4577 | .59049 | 3.4297 | .64235 | 3.4425 | .61853 |  |
| **5. Use of mouth rinse** |  |  |  |  |  |  | 0.529 |
| More than once a day | 3.3636 | .73499 | 3.3505 | .63190 | 3.4775 | .50064 |  |
| Once a day | 3.4649 | .42628 | 3.4566 | .48653 | 3.4026 | .61392 |  |
| Rarely/Never | 3.4673 | .62582 | 3.4514 | .67990 | 3.4425 | .61853 |  |
| **6. Use of chewing gum** |  |  |  |  |  |  | 0.436 |
| More than once a day | 3.4649 | .56516 | 3.3623 | .60326 | 3.4262 | .58073 |  |
| Once a day | 3.4730 | .62564 | 3.4082 | .67074 | 3.4339 | .65221 |  |
| Rarely/Never | 3.3467 | .66397 | 3.5337 | .57791 | 3.4688 | .61339 |  |
| **7. Frequency of sugar intake** |  |  |  |  |  |  | **0.031** |
| More than once a day | 3.3718 | .65476 | 3.3885 | .62457 | 3.3800^a^ | .63910 |  |
| Once a day | 3.5213 | .53222 | 3.5081 | .54389 | 3.5145^b^ | .53693 |  |
| Rarely/Never | 3.7083 | .27722 | 3.4072 | .75187 | 3.4875 | .67051 |  |
| **8. Frequency of Soft Drinks** |  |  |  |  |  |  | **0.001** |
| More than once a day | 3.2179 | .72110 | 3.3551 | .60999 | 3.3032^a^ | .65573 |  |
| Once a day | 3.5188 | .52638 | 3.4731 | .60530 | 3.4974^b^ | .56371 |  |
| Rarely/Never | 3.5267 | .54160 | 3.5018 | .63236 | 3.5148^c^ | .58542 |  |

**^ Within columns, variables with superscripts (^a,b,c^) are statistically significant at 0.05 level (t-test)**

*** Variables with significant p-values are in bold**

**Table S5. Total OHRQoL scores of all the oral behaviours in both genders of 12-14 years aged students (N=607), Kuwait, 2020**

| **Total OHRQoL Score** | | | | | | | |
| --- | --- | --- | --- | --- | --- | --- | --- |
| **Oral Behaviors/Symptoms** | **Girls** | | **Boys** | | **Both Genders** | | **p-value** |
|  | **Mean** | **SD** | **Mean** | **SD** | **Mean** | **SD** |  |
| **1. Last dental visit** |  |  |  |  |  |  | 0.817 |
| < 1 year | 3.1847 | .51211 | 3.1134 | .59440 | 3.1509 | .55288 |  |
| 1-2 years | 3.1379 | .60493 | 3.0286 | .67222 | 3.0758 | .64378 |  |
| > 2 two years | 3.1380 | .63256 | 3.1309 | .52032 | 3.4878 | .52871 |  |
| **2. Pain** |  |  |  |  |  |  | **0.037** |
| Never | 3.3821 | .56797 | 3.4167 | .55228 | 3.4009^a^ | .55625 |  |
| Less than a year | 3.1107 | .52766 | 2.9719 | .63157 | 3.0406^b^ | .58564 |  |
| 1-2 years | 3.2350 | .53404 | 3.0918 | .42842 | 3.0406 | .58564 |  |
| More than 2 years | 3.0820 | .60365 | 3.2206 | .52713 | 3.1687 | .55575 |  |
| **3. Brushing Frequency** |  |  |  |  |  |  | 0.162 |
| More than once a day | 3.1934 | .55187 | 3.2006 | .56596 | 3.1960 | .55582 |  |
| Once a day | 3.1643 | .52377 | 3.0500 | .65104 | 3.0938 | .60645 |  |
| Rarely/Never | 2.9028 | .58285 | 3.0112 | .55598 | 2.9912 | .55864 |  |
| **4. Dental flossing** |  |  |  |  |  |  | 0.918 |
| More than once a day | 3.1146 | .51168 | 3.0569 | .63997 | 3.0826 | .57912 |  |
| Once a day | 3.1783 | .60808 | 3.0306 | .57172 | 3.1170 | .59465 |  |
| Rarely/Never | 3.1694 | .53490 | 3.1126 | .60054 | 3.1385 | .57154 |  |
| **5. Use of mouth rinse** |  |  |  |  |  |  | 0.962 |
| More than once a day | 3.1576 | .61083 | 3.0551 | .61789 | 3.0826 | .57912 |  |
| Once a day | 3.1860 | .46583 | 3.1204 | .44926 | 3.1170 | .59465 |  |
| Rarely/Never | 3.1724 | .56435 | 3.0925 | .66666 | 3.1385 | .57154 |  |
| **6. Use of chewing gum** |  |  |  |  |  |  | 0.977 |
| More than once a day | 3.1721 | .52217 | 3.0126 | .58641 | 3.1106 | .55205 |  |
| Once a day | 3.1698 | .59842 | 3.0912 | .64000 | 3.1215 | .62360 |  |
| Rarely/Never | 3.1522 | .56773 | 3.2057 | .55638 | 3.1860 | .55890 |  |
| **7. Frequency of sugar intake** |  |  |  |  |  |  | **0.001** |
| More than once a day | 3.0637 | .58431 | 3.0438 | .57986 | 3.0538^a^ | .58113 |  |
| Once a day | 3.2965 | .47917 | 3.1793 | .54382 | 3.2382^b^ | .51424 |  |
| Rarely/Never | 3.4246 | .34115 | 3.1153 | .75279 | 3.1955 | .68138 |  |
| **8. Frequency of Soft Drinks** |  |  |  |  |  |  | **0.006** |
| More than once a day | 2.9818 | .65123 | 2.9505 | .60953 | 2.9623^a^ | .62383 |  |
| Once a day | 3.2192 | .46480 | 3.1605 | .53459 | 3.1915^b^ | .49826 |  |
| Rarely/Never | 3.2478 | .52490 | 3.2340 | .61205 | 3.2413^c^ | .56615 |  |

**^ Within columns, variables with superscripts (^a,b,c^) are statistically significant at 0.05 level (t-test)**

*** Variables with significant p-values are in bold**
